# Inpatient and outpatient treatment patterns of cancer-associated thrombosis in the United States

**DOI:** 10.1007/s11239-019-02032-3

**Published:** 2020-01-18

**Authors:** J. D. Guo, P. Hlavacek, T. Poretta, G. Wygant, D. Lane, M. Gorritz, X. Wang, C. C. Chen, R. L. Wade, X. Pan, J. Rajpura, B. Stwalley, L. Rosenblatt

**Affiliations:** 1 Bristol-Myers Squibb, 3401 Princeton Pike, Lawrence Township, Lawrenceville, NJ 08648 USA; 2 Pfizer, Inc., New York, NY USA; 3 IQVIA Inc, Plymouth Meeting, PA USA

**Keywords:** Cancer, Direct oral anticoagulants, Low molecular weight heparin, Warfarin, Venous thromboembolism, Cancer associated thrombosis

## Abstract

Low molecular weight heparins (LMWHs) and direct oral anticoagulants (DOACs) are among the recommended treatment options for cancer-associated thrombosis (CAT) in the 2019 National Comprehensive Care Network guidelines. Little is known about the current utilization of DOACs in CAT patients, particularly on the inpatient to outpatient therapy transition. This study assessed real-world treatment patterns of CAT in hospital/ED in adult cancer patients (≥ 18 years) diagnosed with CAT during a hospital visit in IQVIA’s Hospital Charge Data Master database between July 1, 2015 and April 30, 2018, and followed their outpatient medical and pharmacy claims to evaluate the initial inpatient/ED and outpatient anticoagulants received within 3 months post-discharge. Results showed that LMWH and unfractionated heparin (UFH) were the most common initial inpatient/ED CAT treatments (35.2% and 27.4%, respectively), followed by DOACs (9.6%); 20.8% of patients received no anticoagulants. Most DOAC patients remained on DOACs from inpatient/ED to outpatient settings (71.4%), while 24.1%, 43.5%, and 0.1% of patients treated with LMWH, warfarin, or UFH respectively, remained on the same therapy after discharge. In addition, DOACs were the most common initial post-discharge outpatient therapy. Outpatient treatment persistence and adherence appeared higher in patients using DOACs or warfarin versus LMWH or UFH. This study shows that DOACs are used as an inpatient/ED treatment option for CAT, and are associated with less post-discharge treatment switching and higher persistence and adherence. Further research generating real-world evidence on the role of DOACs to help inform the complex CAT clinical treatment decisions is warranted.

**Electronic supplementary material:**

The online version of this article (10.1007/s11239-019-02032-3) contains supplementary material, which is available to authorized users.

## Highlights

- LMWH and UFH were the most common initial anticoagulants used to treat CAT during an inpatient or ED visit, while DOACs were the most common initial treatments observed after discharge.
- Patients who initiated anticoagulant therapy using DOACs during an inpatient or ED visit were likely to remain on DOACs after discharge, while patients who initiated therapy using other anticoagulants were more likely to switch therapy classes after discharge.
- Patients treated with DOACs or warfarin in the outpatient setting had better treatment persistence and higher adherence than patients treated with LMWH and UFH.
- Additional real-world studies are warranted to compare clinical outcomes, such as recurrent VTE and major bleeding, which will help guide clinical decisions in this area.

## Introduction

Cancer-associated thrombosis (CAT), also known as cancer-associated venous thromboembolism (VTE) [1–3], is the second most common cause of death in cancer patients [4]. Common forms of VTE are deep vein thrombosis (DVT) and pulmonary embolism (PE) [5, 6], which are more likely to occur in cancer patients than in the general population [4]. Risk of developing VTE in cancer patients is four to seven times higher than in patients without cancer [2, 7]. Risk factors for CAT include chemotherapy treatment, certain hormone therapies, surgical interventions, immobilization, and cancer types [4, 8, 9].

Complications like increased risk of recurrent VTE makes the management of CAT complex [10]. Several anticoagulant treatment options are indicated for CAT. Parenteral therapies include low-molecular-weight heparins (LMWH) such as dalteparin, and unfractionated heparin (UFH). Oral therapy options include vitamin K antagonists (e.g., warfarin), and direct-acting oral anticoagulants (DOACs: apixaban, betrixaban, dabigatran, edoxaban and rivaroxaban).

Recent clinical trial data of DOACs for the treatment of CAT have been promising, showing lower rates of VTE recurrence in patients treated with DOACs compared to LMWHs [11, 12]. The ADAM-VTE [11] and SELECT-D [12] trials of apixaban and rivaroxaban, respectively, versus dalteparin in CAT patients showed that DOACs had significantly lower rates of VTE recurrence than the LMWH comparator.

In light of favorable clinical trial results, treatment guidelines for CAT are evolving; the 2019 National Comprehensive Cancer Network (NCCN) guidelines for the treatment of CAT was the first to recommend DOACs. Per the 2019 NCCN guidelines, recommended CAT treatments include LMWH, UFH, or rivaroxaban as monotherapy options; apixaban is recommended as an alternative for patients who refuse or have compelling reasons to avoid LMWH [13].

There is little real-world data on anticoagulant treatment patterns in CAT patients. Most studies report treatments observed either in the hospital or outpatient setting [14, 15]. The transition of anticoagulant treatment from the inpatient to outpatient setting was reported in one abstract [16]. The objectives of this study were to (1) describe the demographic and clinical characteristics of patients with CAT in the US; and (2) understand real-world anticoagulant treatment patterns observed in the inpatient hospital or emergency department (ED) and post-discharge/outpatient settings.

## Methods

### Study design and data sources

This retrospective cohort study utilized three real-world databases: (1) IQVIA Hospital Charge Data Master (CDM) database, (2) IQVIA Patient Centric Pharmacy Claims Database (LRx), and (3) IQVIA Patient Centric Medical Claims Database (Dx) from January 1, 2015 to May 31, 2018.

The CDM database was used to identify anticoagulant therapies received during an inpatient or ED visit. It is comprised of data collected from hospital operational files from over 650 non-federal, acute-care short-stay hospitals. Data elements include all inpatient and outpatient encounters with detailed information on diagnoses, procedures, treatment, and patient and hospital characteristics.

The LRx database was used to identify anticoagulants received from outpatient pharmacies. It consists of pharmacy claims for dispensed prescriptions collected from pharmacies covering approximately 90% of all dispensed prescriptions from US retail pharmacies and over 1.4 billion prescriptions per year, representing claims from all payer types. The Dx database was used to identify anticoagulant treatments administered during outpatient physician office visits. It is composed of approximately 1 billion outpatient medical claims per year submitted by over 860,000 practitioners in the US.

In compliance with the Health Insurance Portability and Accountability Act (HIPAA), patient data were de-identified and, therefore, informed consent and institutional review board (IRB) review were unnecessary.

### Study population

**Fig. 1:**
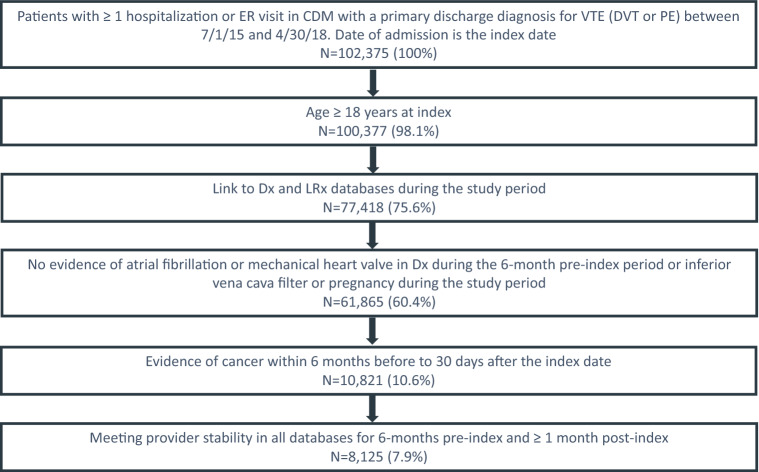
Patient selection flow chart

Patients with CAT were identified during an inpatient or ED visit in CDM between 7/1/2015 and 4/30/2018. Adult patients (age ≥ 18) with a hospital admission or ED visit with primary discharge diagnosis of DVT or PE (an approach that has been shown to have positive predictive value for identifying VTE of 95%; 95% CI 93–97) [17, 18] were included. The first eligible inpatient or ED visit was defined as the index hospital visit and the admission date as the index date. Patients were linked to LRx and Dx, and had evidence of cancer during the 6-months prior to, on, or within 30 days after the index date. Evidence of cancer was defined as (1) ≥ 1 inpatient claim with a cancer diagnosis; (2) ≥ 2 outpatient medical claims with a diagnosis code for the same cancer type; or (3) ≥ 1 outpatient medical claim with a cancer diagnosis and ≥ 1 medical or pharmacy claim for cancer treatment (chemotherapy, biologic treatment, cancer-related hormone therapy, radiation, or cancer-related surgery). Diagnosis codes were identified in CDM and Dx using International Classification of Diseases, Ninth/Tenth Revision, Clinical Modification (ICD-9/10-CM) codes. Cancer treatments were identified in Dx and LRx using Current Procedural Terminology (CPT) codes, Healthcare Common Procedure Coding Systems (HCPCS) Level II codes, and/or National Drug Codes (NDCs). Cancer-related surgery was captured in CDM using CPT codes. Patients with atrial fibrillation/flutter or mechanical heart value during the 6-months before or during the index hospital visit; or inferior vena cava filter or pregnancy during the study period were excluded.  Patients had 6 months of data availability before the index date and ≥ 1 month of follow-up after the discharge date (Fig. 1). Baseline characteristics were assessed during the 6-month pre-index period and on the index date. Details are provided in Table 1.

### Anticoagulant treatment during index hospital visit and transition following discharge

Anticoagulants used during the index hospital visit were captured from billing descriptions in CDM. The index CAT treatment was defined as the first anticoagulant observed. Six treatment groups were formed: (1) DOACs (apixaban, edoxaban, dabigatran, rivaroxaban), (2) LMWH (dalteparin sodium, enoxaparin sodium, fondaparinux sodium, tinzaparin sodium), (3) warfarin (warfarin sodium), (4) UFH (heparin sodium), (5) thrombolytic agents (alteplase, urokinase), and (6) no anticoagulant treatment. If LMWH or UFH were observed on the same date as an oral anticoagulant, the patient was grouped into the specified oral anticoagulant subgroup (e.g., a patient with LMWH and a DOAC on the same date was classified as a DOAC patient). If LMWH and UFH were observed on the same date, the patient was grouped into the LMWH subgroup.

The first outpatient anticoagulant received within 3 months after discharge was captured from the Dx and LRx databases in patients with ≥ 3 months of continuous follow-up after discharge. Sensitivity analyses were conducted in patients with ≥ 1 and ≥ 6 months of follow-up. Results of sensitivity analyses are available in supplemental tables and figures.

### Post-discharge anticoagulant treatment patterns

Outpatient anticoagulant treatment patterns were evaluated in patients with ≥ 3 months of follow-up after discharge and ≥ 3 months of follow-up after initiation of the outpatient therapy. Discontinuation, persistence, and adherence were measured. Discontinuation was defined as a gap > 60 days between the end of days’ supply of a prescription and the next dispensing date of any drug in the same treatment group or as a switch to a new treatment group; discontinuation date was defined as the dispensing date + days’ supply for the prescription before the gap. Persistence was defined as remaining on therapy with no gaps > 60 days between the end of days’ supply for a prescription to the next dispensing date of any drug in the same treatment group. The proportion of patients with persistence to the initial outpatient treatment of ≥ 3 months was reported. Adherence was assessed using medication possession ratio (MPR), calculated as the sum of the days’ supply of all claims occurring before the discontinuation date divided by the number of days between the treatment start date and discontinuation date, and capped at 100%. Adherence was defined as MPR ≥ 80%.

### Statistical analysis

This study was descriptive in nature. Mean and standard deviation (SD) were presented as measures of central tendency and variance for continuous variables. Frequency (N) and percentage (%) of patients in each cohort were reported for categorical variables. All analyses were conducted using SAS version 9.4 (Cary, NC, USA).

## Results

### Patient baseline characteristics

In total 8125 patients with CAT identified during an inpatient or ED visit were included (Fig. 1). Mean age was 65.6 years (SD = 13.0), 46.7% were male, and average length of stay of the index hospital visit was 4 days (SD = 4.0). Use of prior CAT treatments was observed in 60.2% of patients, and 8.0% had a prior CAT diagnosis. Common comorbidities included hypertension (61.3%) and dyslipidemia (39.7%). Lung (18.5%) and breast (14.5%) cancer were the most common primary cancer types. Almost half of patients had no oncology treatment (48.6%), and 39.5% had chemotherapy (Table 1).

**Table 1:** Baseline patient demographic and clinical characteristics stratified by index CAT treatment (n = 8125)

	Total	DOACs	LMWH	Warfarin	UFH	Thrombolytic agent	No anticoagulant treatment
	N = 8125	N = 730	N = 2932	N = 428	N = 2323	N = 122	N = 1590
Age^a^, mean ± SD	65.6 ± 13.0	66.4 ± 12.9	65.2 ± 13.3	67.5 ± 12.6	66.1 ± 12.6	62.9 ± 13.5	65.2 ± 13.3
Male^a^	3793 (46.7%)	363 (49.7%)	1298 (44.3%)	212 (49.5%)	1123 (48.3%)	61 (50.0%)	736 (46.3%)
Geographic region^a^, n (%)							
Northeast	302 (3.7%)	17 (2.3%)	109 (3.7%)	19 (4.4%)	102 (4.4%)	1 (0.8%)	54 (3.4%)
Midwest	489 (6.0%)	43 (5.9%)	143 (4.9%)	28 (6.5%)	165 (7.1%)	12 (9.8%)	98 (6.2%)
South	2454 (30.2%)	221 (30.3%)	957 (32.6%)	110 (25.7%)	659 (28.4%)	43 (35.2%)	464 (29.2%)
West	496 (6.1%)	43 (5.9%)	159 (5.4%)	47 (11.0%)	114 (4.9%)	6 (4.9%)	127 (8.0%)
Unknown	4384 (54.0%)	406 (55.6%)	1564 (53.3%)	224 (52.3%)	1283 (55.2%)	60 (49.2%)	847 (53.3%)
Insurance type^a^, n (%)							
Cash	6 (0.1%)	0 (0.0%)	0 (0.0%)	0 (0.0%)	3 (0.1%)	0 (0.0%)	3 (0.2%)
Medicaid	154 (1.9%)	13 (1.8%)	55 (1.9%)	8 (1.9%)	35 (1.5%)	2 (1.6%)	41 (2.6%)
Medicare	2735 (33.7%)	277 (37.9%)	955 (32.6%)	179 (41.8%)	773 (33.3%)	35 (28.7%)	516 (32.5%)
Third Party	2193 (27.0%)	200 (27.4%)	816 (27.8%)	101 (23.6%)	623 (26.8%)	29 (23.8%)	424 (26.7%)
Unknown	3037 (37.4%)	240 (32.9%)	1106 (37.7%)	140 (32.7%)	889 (38.3%)	56 (45.9%)	606 (38.1%)
Type of index CAT^a^, n (%)							
DVT	3743 (46.1%)	427 (58.5%)	1232 (42.0%)	218 (50.9%)	782 (33.7%)	47 (38.5%)	1037 (65.2%)
PE	4382 (53.9%)	303 (41.5%)	1700 (58.0%)	210 (49.1%)	1541 (66.3%)	75 (61.5%)	553 (34.8%)
Setting of CAT diagnosis^a^, n (%)							
Inpatient	5728 (70.5%)	325 (44.5%)	2245 (76.6%)	344 (80.4%)	2171 (93.5%)	117 (95.9%)	526 (33.1%)
ED	2397 (29.5%)	405 (55.5%)	687 (23.4%)	84 (19.6%)	152 (6.5%)	5 (4.1%)	1064 (66.9%)
Prior CAT diagnosis^b^, n (%)	652 (8.0%)	52 (7.1%)	192 (6.5%)	46 (10.7%)	176 (7.6%)	20 (16.4%)	166 (10.4%)
Prior CAT treatment^b^, n (%)	4893 (60.2%)	389 (53.3%)	1823 (62.2%)	251 (58.6%)	1388 (59.8%)	89 (73.%)	953 (59.9%)
Top 5 comorbidities^c^, n (%)							
Diabetes	2023 (24.9%)	155 (21.2%)	710 (24.2%)	123 (28.7%)	641 (27.6%)	35 (28.7%)	359 (22.6%)
Dyslipidemia	3226 (39.7%)	270 (37.0%)	1123 (38.3%)	205 (47.9%)	1025 (44.1%)	46 (37.7%)	557 (35.0%)
Hypertension	4982 (61.3%)	420 (57.5%)	1769 (60.3%)	297 (69.4%)	1524 (65.6%)	74 (60.7%)	898 (56.5%)
Osteoarthritis	2398 (29.5%)	219 (30.0%)	854 (29.1%)	128 (29.9%)	721 (31.0%)	28 (23.0%)	448 (28.2%)
Smoking or history of smoking	2581 (31.8%)	196 (26.8%)	919 (31.3%)	116 (27.1%)	807 (34.7%)	43 (35.2%)	500 (31.4%)
CCI^c^, mean ± SD	4.4 (2.9)	3.9 (2.8)	4.4 (2.8)	4.0 (2.9)	4.3 (3.0)	4.8 (2.6)	4.2 (2.9)
Top 5 cancer types^d^(n, %)							
Malignant neoplasm of bronchus and lung	1500 (18.5%)	102 (14.%)	570 (19.4%)	54 (12.6%)	487 (21.%)	16 (13.1%)	271 (17.%)
Malignant neoplasm of breast	1176 (14.5%)	129 (17.7%)	445 (15.2%)	57 (13.3%)	287 (12.4%)	17 (13.9%)	241 (15.2%)
Malignant neoplasm of testis	724 (8.9%)	98 (13.4%)	216 (7.4%)	50 (11.7%)	186 (8.0%)	16 (13.1%)	158 (9.9%)
Malignant neoplasm of colon	625 (7.7%)	58 (7.9%)	217 (7.4%)	27 (6.3%)	190 (8.2%)	11 (9.0%)	122 (7.7%)
Malignant neoplasm of pancreas	470 (5.8%)	31 (4.2%)	191 (6.5%)	14 (3.3%)	141 (6.1%)	9 (7.4%)	84 (5.3%)
Metastatic status^d^(n, %)							
Metastatic	150 (1.8%)	9 (1.2%)	66 (2.3%)	15 (3.5%)	43 (1.9%)	1 (0.8%)	16 (1.0%)
Non-metastatic	7975 (98.2%)	721 (98.8%)	2866 (97.7%)	413 (96.5%)	2280 (98.1%)	121 (99.2%)	1574 (99.0%)
Oncology treatment history^d^(n, %)							
No pre-index oncology treatment	3951 (48.6%)	370 (50.7%)	1340 (45.7%)	240 (56.1%)	1133 (48.8%)	52 (42.6%)	816 (51.3%)
I-O therapy (biologic therapy)	163 (2.0%)	13 (1.8%)	56 (1.9%)	6 (1.4%)	54 (2.3%)	1 (0.8%)	33 (2.1%)
Chemotherapy	3208 (39.5%)	263 (36.%)	1286 (43.9%)	129 (30.1%)	911 (39.2%)	59 (48.4%)	560 (35.2%)
Cancer-related hormone therapy	709 (8.7%)	75 (10.3%)	242 (8.3%)	43 (10.0%)	189 (8.1%)	11 (9.0%)	149 (9.4%)
Radiation therapy	652 (8.0%)	39 (5.3%)	246 (8.4%)	27 (6.3%)	207 (8.9%)	14 (11.5%)	119 (7.5%)
Surgery	416 (5.1%)	56 (7.7%)	128 (4.4%)	27 (6.3%)	100 (4.3%)	4 (3.3%)	101 (6.4%)
Year of index date (n, %)							
2015	1296 (16.0%)	87 (11.9%)	519 (17.7%)	134 (31.3%)	323 (13.9%)	15 (12.3%)	218 (13.7%)
2016	2847 (35.0%)	257 (35.2%)	1034 (35.3%)	160 (37.4%)	779 (33.5%)	43 (35.2%)	574 (36.1%)
2017	3104 (38.2%)	296 (40.5%)	1078 (36.8%)	110 (25.7%)	935 (40.2%)	49 (40.2%)	636 (40.0%)
2018	878 (10.8%)	90 (12.3%)	301 (10.3%)	24 (5.6%)	286 (12.3%)	15 (12.3%)	162 (10.2%)
Length of stay (LOS), mean ± SD	4.0 (4.0)	2.2 (2.4)	4.2 (3.8)	4.5 (3.9)	5.5 (4.3)	6.7 (7.2)	2.1 (2.6)

^a^Measure assessed on the index date

^b^Measure assessed during the 6-month pre-index period, not including the index date

^c^Measure assessed during the 6-month pre-index period, including the index date

^d^Measure assessed during the 6-month pre-index period through 30-days post-index

### Transition of CAT treatment from the inpatient/ED setting to the outpatient setting

**Fig. 2:**
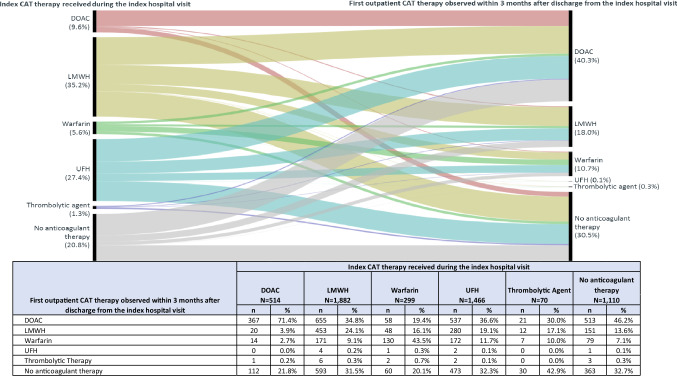
Sequence of the initial anticoagulant therapies received during the index hospital visit and within 3 months after discharge in patients with at least 3 months of follow-up (n = 5341). Values provided are the percentage of patients who were treated with the specified therapies during the index hospital visit (left-hand bar), and the initial treatment received in the outpatient setting within 3 months after discharge (right-hand bar). The shaded pathways represent the proportion of patients who flow from the specified hospital treatment to the specified outpatient treatments

A total of 5341 patients (65.7%) had ≥ 3 months of post-discharge follow-up. LMWH and UFH were the most common initial anticoagulants during the index hospital visit (35.2% and 27.4%, respectively), followed by DOACs (9.6%). Anticoagulant therapy was not observed in 20.8% of patients (Fig. 2).

Over 70% of patients who started treatment with DOACs (n = 514) remained on DOACs after discharge (71.4%). Conversely, only 24.1% of patients treated with LMWH and 43.5% treated with warfarin during the index hospital visit remained on the index CAT treatment after discharge (Fig. 2). Among patients with no anticoagulant treatment during the index hospital visit (N = 1110), 46.2% received DOACs, 13.6% received LMWH, 7.1% received warfarin, and 32.7% did not receive outpatient treatment within 3 months after discharge.

Within 3 months after discharge, DOACs were most frequently used in the outpatient setting (40.3%), followed by LMWH (18.0%) and warfarin (10.7%); nearly one-third of patients (30.5%) had no outpatient anticoagulants (Fig. 2). Treatment transition patterns from the index hospital visit to the outpatient setting were similar with 1- and 6-month follow-up periods (Supplemental Figs. 3 and 4).

### Post-discharge outpatient anticoagulant treatment patterns

Among the 5341 patients with ≥ 3 months of follow-up after discharge, a total of 2243 patients had a claim for outpatient anticoagulant therapy within 3 months after discharge, and had ≥ 3 months of follow-up after outpatient treatment initiation (Table 2). Overall, 23.3% of patients discontinued the outpatient anticoagulant therapy within 3 months, with a lower rate of discontinuation in patients treated with DOACs (12.2%) or warfarin (17.2%) compared to LMWH (47.0%) and UFH (50.0%). A higher proportion of patients treated with DOACs and warfarin were persistent to the initial outpatient anticoagulant at 3 months after treatment initiation compared to those treated with LMWH and UFH (DOACs: 87.8%; LMWH: 53.0%; warfarin: 82.8%; UFH: 50.0%). Oral anticoagulants had higher adherence than parenteral anticoagulants; 88.8% of patients treated with DOACs and 89.0% of warfarin patients had MPR ≥ 80% compared to 76.4% of patients treated with LMWH (Table 2). Similar trends were observed in patients treated with anticoagulants within 1- and 6-months of discharge from the index hospital visit (Supplemental Tables 3 and 4).

**Table 2:** Treatment patterns of the initial post-discharge anticoagulant treatment received within 3 months after discharge among CAT patients with ≥ 3 months of follow-up after discharge and ≥ 3 months of follow-up after initiation of the outpatient treatment (n = 2243)

	Total N = 2243	DOACs N = 1300	LMWH N = 526	Warfarin N = 408	UFH N = 6	Thrombolytic therapy N = 3
Patients with discontinuation within 3 months of treatment initiation^a^(n,%)	523 (23.3%)	159 (12.2%)	247 (47.0%)	70 (17.2%)	3 (50.0%)	3 (100.0%)
Persistence to therapyat 3 months after treatment initiation^b^(n,%)	1720 (76.7%)	1141 (87.8%)	279 (53.0%)	338 (82.8%)	3 (50.0%)	0 (0.0%)
MPR^c^, mean ± SD	0.9 (0.1)	0.9 (0.1)	0.9 (0.2)	0.9 (0.1)	0.4 (0.3)	1.0 (0.0)
Adherence (MPR ≥ 0.80; n, %)	1923 (85.7%)	1154 (88.8%)	402 (76.4%)	363 (89.0%)	0 (0.0%)	3 (100.0%)

1631 patients did not have evidence of anticoagulant treatment within 3 months after discharge; 1467 patients had outpatient anticoagulant therapy, but had less than 3 months of follow-up after outpatient treatment initiation.

^a^Discontinuation is defined as a gap of > 60 days between end of days' supply for a prescription to the next dispensing date of a drug in the same treatment group, or as a switch to a new treatment group. Last date of days’ supply before this gap is the discontinuation date

^b^Persistence to therapy is defined as remaining on therapy with no gaps > 60 days between the end of days’ supply for a prescription to the next fill date of any drug in the same treatment group

^c^MPR is defined as the sum of days’ supply for all claims prior to the discontinuation date (i.e., while a patient is on therapy)

## Discussion

This study is the first publication that provides a recent look at anticoagulant treatment patterns using real-world data after the release of the 2019 NCCN guideline supporting use of DOACs as an alternative to LMWH in cancer patients [13] and promising clinical trial results showing lower rates of recurrent VTE in patients treated with DOACs compared to LMWHs [11, 12]. It examined the use of anticoagulant therapy in CAT patients during an inpatient or ED visit, and allowed for longitudinal tracking of CAT patients from the initial hospital visit to the post-discharge outpatient setting to evaluate transition of therapy between settings.

Consistent with literature [14, 15], LMWH and UFH were the most common anticoagulants used to treat CAT during an inpatient or ED visit (35.2% and 27.4%, respectively; Fig. 2). Results also suggests that, consistent with NCCN guidelines [13], DOACs are being used as first-line CAT treatment in the hospital setting. DOACs were the most commonly observed post-discharge anticoagulant treatment (46.2%), and most patients who started DOACs in the hospital setting stayed on DOACs after discharge (71.4%) (Fig. 2). In addition, this study observed that patients treated with outpatient oral anticoagulants stayed on their anticoagulant therapy longer and with better adherence, compared to parenteral anticoagulant therapy. The proportion of patients with persistence ≥ 3 months after outpatient treatment initiation ranged from 82.8–87.8% for oral and 50.0–53.0% for injectable anticoagulants; 89% of patients treated with oral anticoagulants and 0–76.4% of patients treated with injectable anticoagulants had high adherence (Table 2). Khorana et al. reported similar findings in their 2017 publication; patients initiating LMWH had shorter persistence and were more likely to discontinue than patients treated with DOACs or warfarin [14].

Roughly 20% of CAT patients in this study did not receive anticoagulant treatment during the index hospital visit (Fig. 2). As cancer patients may present themselves to the hospital with other complex clinical conditions, it’s possible that physicians prioritized treating more urgent conditions over CAT or had other compelling clinical justification not to initiate anticoagulant therapy. Furthermore, patients may have received non-pharmacological interventions (i.e., thrombolysis) which were not assessed. Approximately 31% of patients who were treated for their CAT in the hospital did not receive further outpatient anticoagulant therapy in the 3 months following discharge (Fig. 2). This result is in line with a prior claims database study published in 2015 which found that 30% of CAT patients were not treated with anticoagulants [19]. Further research exploring reasons for not receiving CAT is warranted.

This study has several strengths. This is the first study to describe anticoagulant treatment patterns in CAT patients from the inpatient through the post-discharge outpatient setting. The use of a large hospital database allowed for a diverse cohort of patient types and hospital settings, and the ability to capture health care records for patients regardless of health plan or payer type, supporting the generalizability of the findings. Furthermore, the availability of outpatient pharmacy and medical claims allowed for an assessment of treatment changes between the inpatient/ED and outpatient settings, as well as adherence and persistence of the outpatient treatment.

As with all observational research, there are inherent limitations to this study. The data were collected for billing purposes rather than research, and key information about treatment decisions (likely influenced by clinical risk factors), physician preference, patient preference or insurance coverage issues, are lacking. The LRx and Dx databases are open-source data and might not capture complete patient activity. Finally, clinical endpoints, such as post-discharge recurrent VTE or major bleeding adverse events, were not evaluated due to the limited sample size having sufficient follow-up.

## Conclusion

While LMWH was most commonly used to treat CAT during an inpatient/ED visit, patients were also treated with DOACs in this setting, consistent with current treatment guidelines and prior recommendations. Furthermore, patients treated with DOACs in the hospital were less likely to switch to a different therapy after discharge. Patients treated with oral anticoagulants post-discharge in the outpatient setting had better persistence after treatment initiation and better adherence than patients treated with parenteral anticoagulants. This study adds evidence supporting the use of DOACs to treat CAT. Future real-world studies to evaluate effectiveness and safety of DOACs with longer follow-up and larger sample size are warranted to further inform the complex clinical decisions in patients with CAT.

## Electronic supplementary material

Below is the link to the electronic supplementary material.

Supplementary material 1 (DOCX 15.4 kb)

Supplementary material 2 (DOCX 13.7 kb)

Supplemental Fig. 3 Sequence of the initial anticoagulant therapies received during the index hospital visit and within 1 month after discharge (n = 8125). Values provided are the percentage of patients who were treated with the specified anticoagulants during the index hospital visit (left-hand bar), and the initial anticoagulant received in the outpatient setting within 1 month after discharge (right-hand bar). The shaded pathways represent the proportion of patients who flow from the specified hospital treatment to the specified outpatient treatments. Supplementary material 3 (PPTX 501.5 kb)

Supplemental Fig. 4 Sequence of the initial anticoagulant therapies received during the index hospital visit and within 6 months after discharge in patients with at least 6 months of follow-up (n = 3093). Values provided are the percentage of patients who were treated with the specified therapies during the index hospital visit (left-hand bar), and the initial treatment received in the outpatient setting within 6 months after discharge (right-hand bar). The shaded pathways represent the proportion of patients who flow from the specified hospital treatment to the specified outpatient treatments. Supplementary material 4 (PPTX 285.7 kb)
