# Supplementary material for: Inpatient and outpatient treatment patterns of cancer-associated thrombosis in the United States
Source: J Thromb Thrombolysis. 2020 Jan 18;50(2):386–94. doi: 10.1007/s11239-019-02032-3 (PMC7366581; doi:10.1007/s11239-019-02032-3)
Supplement: Supplementary file 1 — Supplementary material 1 (DOCX 15.4 kb) [file 11239_2019_2032_MOESM1_ESM.docx]

**Supplemental T1.** **Treatment patterns of the initial post-discharge anticoagulant treatment received within 1 month after discharge among CAT patients (n=3,469)**

|  | **Total**  **N=3,469** | **DOAC**  **N=1,943** | **LMWH N=910** | **Warfarin N=601** | **UFH**  **N=9** | **Thrombolytic Therapy**  **N=6** |
| --- | --- | --- | --- | --- | --- | --- |
| **Patients with discontinuation within 1 month of treatment initiation^1^ (n, %)** | 449 (12.9%) | 129 (6.6%) | 228 (25.1%) | 50 (8.3%) | 3 (33.3%) | 3 (50.0%) |
| **Persistence to therapy at 1 month after treatment initiation^2^ (n, %)** | 3,020 (87.1%) | 1,814 (93.4%) | 682 (74.9%) | 551 (91.7%) | 6 (66.7%) | 3 (50.0%) |
| **MPR^3^ (mean, SD)** | 0.9 (0.2) | 0.9 (0.1) | 0.9 (0.2) | 0.9 (0.1) | 0.4 (0.3) | 0.4 (0.5) |
| **Adherent (MPR ≥0.80; n,%)** | 2,889 (83.3%) | 1,686 (86.8%) | 670 (73.6%) | 532 (88.5%) | 1 (11.1%) | 2 (33.3%) |

Note: 3,607 patients did not have evidence of anticoagulant treatment within 1 month after discharge; 1,049 patients had outpatient anticoagulant therapy, but had less than 1 month of follow-up after outpatient treatment initiation.

^1^ Discontinuation is defined as a gap of > 60 days between end of days' supply for a prescription to the next dispensing date. Last date of days’ supply before this gap is the discontinuation date.

^2^ Persistence to therapy is defined as remaining on therapy with no gaps > 60 days between the end of days’ supply for a prescription to the next fill date of any drug in the same treatment group.

^3^ MPR is defined as the sum of days’ supply for all claims prior to the discontinuation date (i.e., while a patient is on therapy).

**Supplemental T2.** **Treatment patterns of the initial post-discharge anticoagulant treatment received within 6 months after discharge among CAT patients with ≥6 months of follow-up (n=960)**

|  | **Total**  **N=960** | **DOACs N=564** | **LMWH N=188** | **Warfarin N=205** | **UFH**  **N=3** | **Thrombolytic Therapy**  **N=0** |
| --- | --- | --- | --- | --- | --- | --- |
| **Patients with discontinuation within 6 months of treatment initiation^1^ (n, %)** | 314 (32.7%) | 118 (20.9%) | 113 (60.1%) | 59 (28.8%) | 1 (33.3%) | 0 (0.0%) |
| **Persistence to therapy at 6 months after treatment initiation^2^ (n, %)** | 646 (67.3%) | 446 (79.1%) | 75 (39.9%) | 146 (71.2%) | 2 (66.7%) | 0 (0.0%) |
| **MPR^3^ (mean, SD)** | 0.9 (0.1) | 0.9 (0.1) | 0.9 (0.2) | 0.9 (0.1) | 0.3 (0.3) | - (-) |
| **Adherent (MPR ≥0.80; n,%)** | 846 (88.1%) | 506 (89.7%) | 157 (83.5%) | 185 (90.2%) | 0 (0.0%) | 0 (0.0%) |

Note: 848 patients did not have evidence of anticoagulant treatment within 6 months after discharge; 1,285 patients had outpatient anticoagulant therapy, but had less than 6 months of follow-up after outpatient treatment initiation.

^1^ Discontinuation is defined as a gap of > 60 days between end of days' supply for a prescription to the next dispensing date. Last date of days’ supply before this gap is the discontinuation date.

^2^ Persistence to therapy is defined as remaining on therapy with no gaps > 60 days between the end of days’ supply for a prescription to the next fill date of any drug in the same treatment group.

^3^ MPR is defined as the sum of days’ supply for all claims prior to the discontinuation date (i.e., while a patient is on therapy).
