# Supplementary figures and images for: Inpatient and outpatient treatment patterns of cancer-associated thrombosis in the United States
Source: J Thromb Thrombolysis. 2020 Jan 18;50(2):386–94. doi: 10.1007/s11239-019-02032-3 (PMC7366581; doi:10.1007/s11239-019-02032-3)

## Slide 1
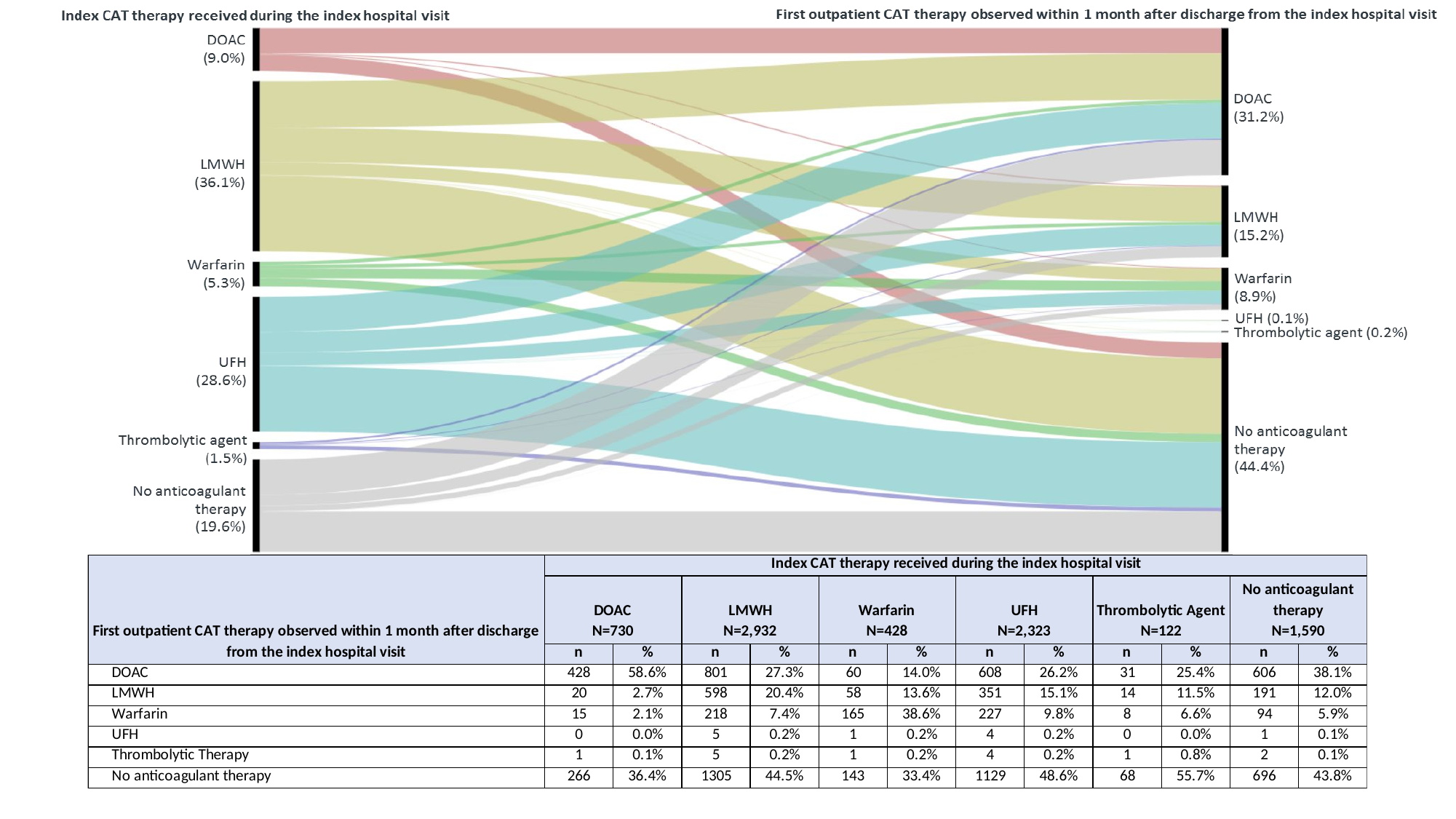

Supplement: Supplementary file 3 — Sequence of the initial anticoagulant therapies received during the index hospital visit and within 1 month after discharge (n = 8125). Values provided are the percentage of patients who were treated with the specified anticoagulants during the index hospital visit (left-hand bar), and the initial anticoagulant received in the outpatient setting within 1 month after discharge (right-hand bar). The shaded pathways represent the proportion of patients who flow from the specified hospital treatment to the specified outpatient treatments. Supplementary material 3 (PPTX 501.5 kb) [file 11239_2019_2032_MOESM3_ESM.pptx]
