# Supplementary material for: Inpatient and outpatient treatment patterns of cancer-associated thrombosis in the United States
Source: J Thromb Thrombolysis. 2020 Jan 18;50(2):386–94. doi: 10.1007/s11239-019-02032-3 (PMC7366581; doi:10.1007/s11239-019-02032-3)
Supplement: Supplementary file 4 — Sequence of the initial anticoagulant therapies received during the index hospital visit and within 6 months after discharge in patients with at least 6 months of follow-up (n = 3093). Values provided are the percentage of patients who were treated with the specified therapies during the index hospital visit (left-hand bar), and the initial treatment received in the outpatient setting within 6 months after discharge (right-hand bar). The shaded pathways represent the proportion of patients who flow from the specified hospital treatment to the specified outpatient treatments. Supplementary material 4 (PPTX 285.7 kb) [file 11239_2019_2032_MOESM4_ESM.pptx]

## Slide 1
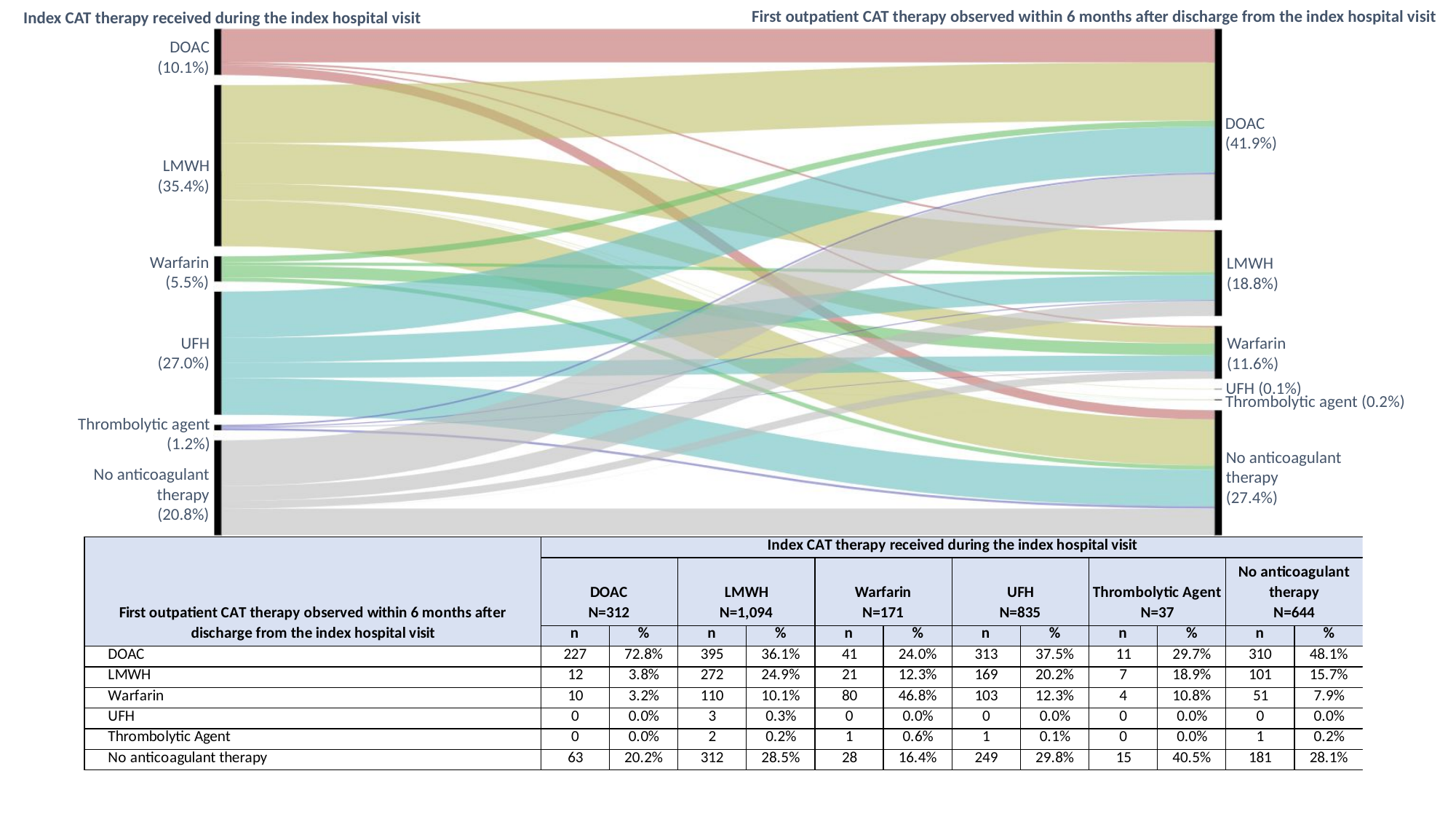

First outpatient CAT therapy observed within 6 months after discharge from the index hospital visit
Index CAT therapy received during the index hospital visit
DOAC
(10.1%)
DOAC
(41.9%)
LMWH
(35.4%)
Warfarin
(5.5%)
LMWH
(18.8%)
UFH
(27.0%)
Warfarin
(11.6%)
UFH (0.1%)
Thrombolytic agent (0.2%)
Thrombolytic agent
(1.2%)
No anticoagulant therapy
(27.4%)
No anticoagulant
therapy
(20.8%)
